# Complete chloroplast genome of *Sinosenecio jishouensis* D.G. Zhang, Ying Liu & Q. E. Yang (Asteraceae), a narrow endemic species in Wuling Mountain Region, China

**DOI:** 10.1080/23802359.2021.1891981

**Published:** 2021-03-18

**Authors:** Qiang Zhou, Jian-Hua Ou-Yang, Jie-Nan Xie, Yao Sun, Ming-Yang Dong

**Affiliations:** a Key Laboratory of Plant Resources Conservation and Utilization, Jishou University, Jishou, Hunan, China; b Guangdong Mercells Cell Biotechnology Co., Ltd, Foshan, Guangdong, China

**Keywords:** Chloroplast genome, *Sinosenecio jishouensis*, Asteraceae, phylogenetic analysis

## Abstract

The first complete chloroplast genome (cp) of *Sinosenecio jishouensis* D.G. Zhang, Ying Liu & Q. E. Yang (Asteraceae) was sequenced and assembled in this study. The cp genome was 151,257 bp in length, including a large single-copy(LSC) region of 83,373 bp, a small single-copy (SSC) region of 18,178 bp, and a pair of inverted repeat (IR) regions of 24,853 bp each. These sequences encoded 134 genes, including 89 protein-coding genes, 37 tRNA genes, and 8 rRNA genes. The phylogenetic analysis based on 18 complete cp sequences revealed that *S. jishouensis* was closely related to *Eclipta prostrata.*

*Sinosenecio jishouensis* D.G. Zhang, Ying Liu & Q. E. Yang 2008 is a perennial herb belonging to Asteraceae (Sinosenecio), which is an extremely small population and narrow endemic plant species to the Wuling mountain region (Zhang et al. 2008; Xiang et al. 2014). And it only lives in a cool, humid habitat and mainly relies on vegetative propagation (Deng et al. 2011), which is probably an important factor limiting its geographic distribution (Zhou et al. 2014). In the present research, we sequenced and assembled the complete chloroplast (cp) genome of *S. jishouensis*, which are beneficial for elucidating evolutionary mechanisms and constructing the phylogeny of Asteraceae species based on cp genome sequences.

Fresh leaves of *S. jishouensis* were collected in Dehang Geological Park (Hunan Province, China; 109°35′49ʺE, 28°21′58ʺN) and were silica-dried and taken to the laboratory until DNA extraction. The voucher specimen was deposited at the herbarium of Jishou University (Qiang Zhou, zhouqiang@jsu.edu.cn) under the voucher number JIU2020ZQ016. High-quality total DNA was extracted from the silica-dried leaf and the whole genome sequencing was conducted by Guangdong Mercells Cell Biotechnology Co., Ltd. (Foshan, China) on the Illumina Hiseq platform. The clean data were assembled by using the program GetOrganelle (Jin et al. 2018), it was annotated using a web-based annotation program GeSeq (https://chlorobox.mpimp-golm.mpg.de/geseq.html) (Tillich et al. 2017) coupled with manual check and adjustment using *Ligularia jaluensis* (NC_039383) as a reference. Finally, the complete cp genome sequence of *S. jishouensis* was deposited in GenBank.

The complete chloroplast genome of *S. jishouensis* (GenBank accession number: MT876597) was 151,257 bp in size, containing a large single-copy (LSC) region of 83,373 bp, a small single-copy (SSC) region of 18,178 bp and a pair of inverted repeat (IR) regions of 24,853 bp. The overall GC content was 37.37% in the whole sequence, while the corresponding values of the LSC, SSC, and IR regions were 35.50%, 30.59%, and 43.00%, respectively. This chloroplast genome contained 134 genes, including 89 protein-coding genes (2 pseudo genes), 37 tRNA genes, and 8 rRNA genes.

A phylogenetic analysis was performed using the complete chloroplast genome of *S. jishouensis* with those from 16 other Asteraceae species and *Daucus carota* (Apiaceae) was used as an outgroup. All of sequences were downloaded from NCBI GenBank, these sequences were aligned with MAFFT (Katoh and Standley 2013), and then the Maximum-likelihood phylogenetic tree was constructed by MEGA 7.0 (Kumar et al. 2016). The phylogenetic tree showed that *S. jishouensis* was closely related to *Eclipta prostrata* (Figure 1).

**Figure 1.**
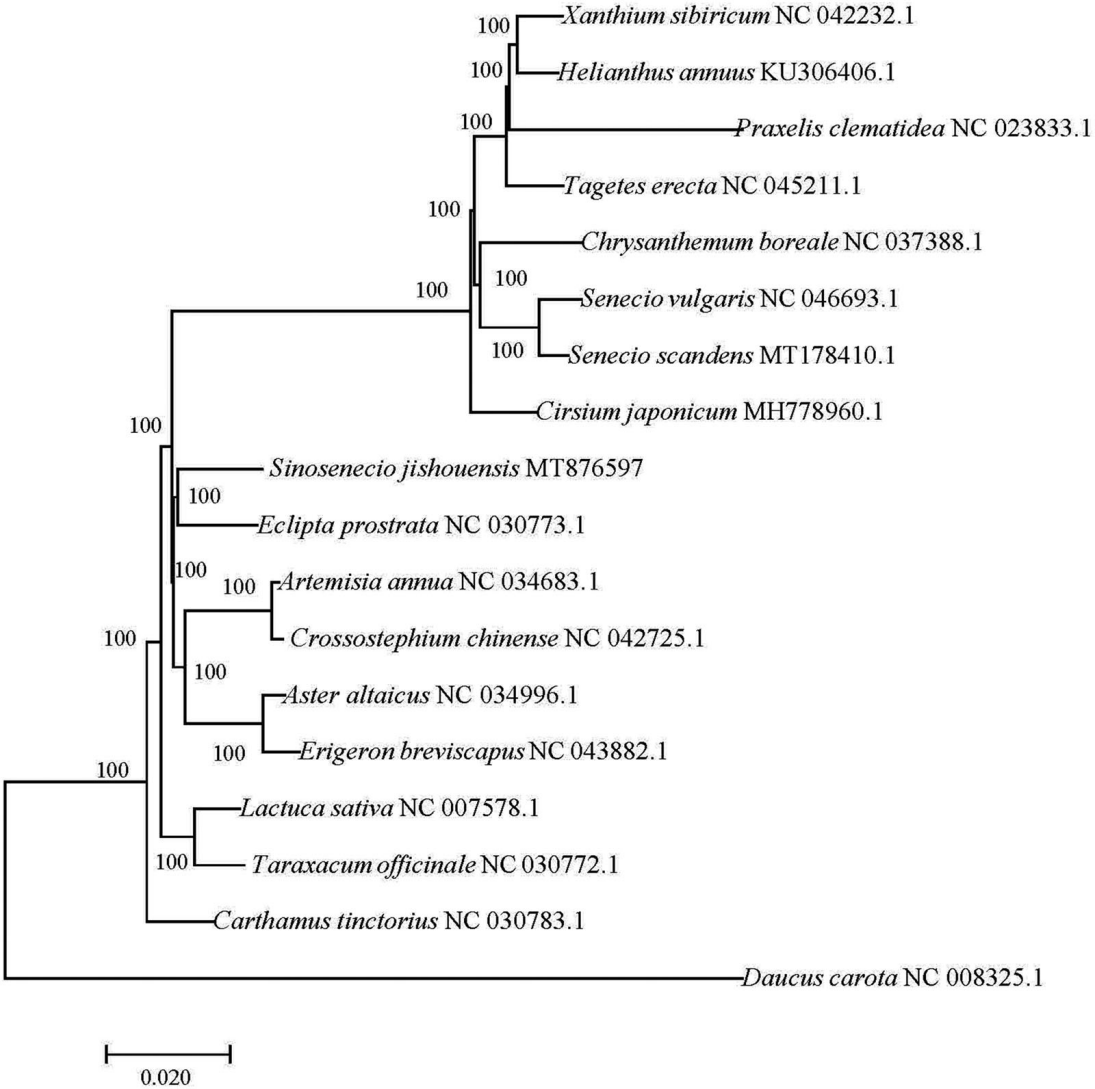
Maximum likelihood phylogenetic tree of 18 species based on whole chloroplast genome data. Bootstrap support values are shown above each branch.

## Data Availability

The genome sequence data that support the findings of this study are openly available in GenBank of NCBI at (https://www.ncbi.nlm.nih.gov/) under the accession no. MT876597. The associated BioProject, SRA, and Bio-Sample numbers are PRJN694336, SRR13528379, and SAMN17517254, respectively.
